# Fiber density of collagen grafts impacts rabbit urethral regeneration

**DOI:** 10.1038/s41598-018-27621-9

**Published:** 2018-07-03

**Authors:** H. M. Larsson, G. Vythilingam, K. Pinnagoda, E. Vardar, E. M. Engelhardt, S. Sothilingam, R. C. Thambidorai, T. Kamarul, J. A. Hubbell, P. Frey

**Affiliations:** 1 Institute of Bioengineering, École Polytechnique Fédérale de Lausanne, Lausanne, Switzerland; 2 Department of Pediatrics, Centre Hospitalier Universitaire Vaudois (CHUV), Lausanne, Switzerland; 3 Department of Surgery, University Malaya, Kuala Lumpur, Malaysia; 4 Tissue Engineering Group, Department of Orthopaedic Surgery, (NOCERAL) University Malaya, Kuala Lumpur, Malaysia; 5 Institute for Molecular Engineering, University of Chicago, Chicago, IL USA

## Abstract

There is a need for efficient and “off-the-shelf” grafts in urethral reconstructive surgery. Currently available surgical techniques require harvesting of grafts from autologous sites, with increased risk of surgical complications and added patient discomfort. Therefore, a cost-effective and cell-free graft with adequate regenerative potential has a great chance to be translated into clinical practice. Tubular cell-free collagen grafts were prepared by varying the collagen density and fiber distribution, thereby creating a polarized low fiber density collagen graft (LD-graft). A uniform, high fiber density collagen graft (HD-graft) was engineered as a control. These two grafts were implanted to bridge a 2 cm long iatrogenic urethral defect in a rabbit model. Histology revealed that rabbits implanted with the LD-graft had a better smooth muscle regeneration compared to the HD-graft. The overall functional outcome assessed by contrast voiding cystourethrography showed patency of the urethra in 90% for the LD-graft and in 66.6% for the HD-graft. Functional regeneration of the rabbit implanted with the LD-graft could further be demonstrated by successful mating, resulting in healthy offspring. In conclusion, cell-free low-density polarized collagen grafts show better urethral regeneration than high-density collagen grafts.

## Introduction

There is a clinical need for efficient and cost-effective grafts in urethral reconstructive surgery. Current surgical techniques require harvesting from autologous sites eg. buccal mucosa and penile skin tissue^1^. This increases the risk of complications, the possible lack of tissue availability and patient discomfort. Certain oral pathologies are contraindications for buccal mucosal graft harvesting^2^. Both cell-free and cell-seeded urethral grafts have been investigated. To date, cell-based grafts have shown better functional results in preclinical large animal models and inconclusive results in subsequent clinical trials^3^. In 2015 and 2017, the clinical outcome of a commercial cell-seeded tissue engineered buccal mucosa product, was published^4,5^. This product requires a buccal biopsy to be taken, followed by *ex-vivo* cell culturing in a GMP facility and a 3-week manufacturing time. The cost-effectiveness and efficacy of these grafts have been debated^6,7^. Therefore, it is unlikely that this approach will become standard clinical practice for the patient with limited financial resources or insurance coverage. To overcome these problems we have targeted the development of a cell-free urethral graft that is cost-effective and adequate also to be used in third world countries.

*In vitro* the mechanical niche is an important parameter to be considered for controlling cell fate. For example, mesenchymal stem cells are known to differentiate into different cell lineages, depending on the substrate stiffness^8^. Previous studies have shown that fluid content or collagen fiber density relates to the stiffness and influenced material properties of the collagen gels^9,10^. The more extracted fluid from the collagen gels, the stiffer the collagen material gets. In this manuscript we address the question if collagen fiber density of a collagen graft influences the functional regeneration of the rabbit urethra? Two different cell-free bovine tubular collagen grafts were manufactured, one with polarized and low collagen fiber density distribution (LD-graft) and one control with uniform and high collagen fiber density distribution (HD-graft). Polarization was achieved by specific compression and the collagen density increased towards the luminal side of the tube. These grafts were implanted in rabbits and were examined post-implantation by histology and immunohistochemistry, and the functional outcome was assessed by voiding cystourethrography and mating.

## Materials and Methods

### Preparation of collagen grafts

**Figure 1:**
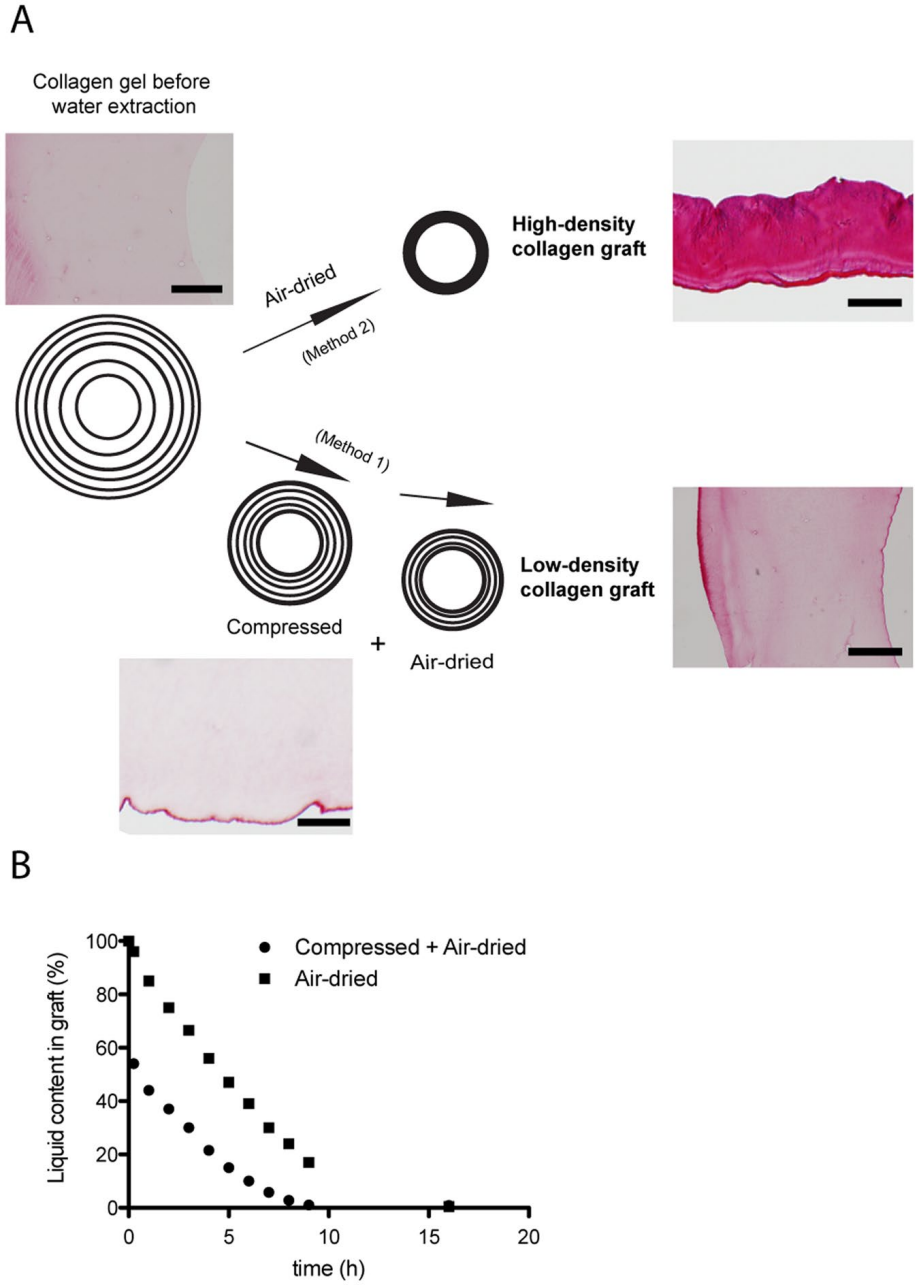
Manufacturing technique of the low-density graft (LD-graft) and high-density graft (HD-graft) (**A**) Two methods were utilized to manufacture the density controlled collagen grafts by monitoring the water content removed either by rolling compression or air-drying. The polarized collagen fiber distribution characteristics of the LD-graft were achieved by utilizing both a rolling compression followed by air-drying. The uniform collagen fiber distribution characteristics of the HD-graft were achieved by only air-drying the collagen. (**B**) By weighing and monitoring the collagen gel during production, we could achieve our polarized (compressed and air-dried: LD-graft) and uniform (only air-dried: HD-graft) collagen fiber distribution characteristics. Note: Images in Fig. 1A are Sirius red stained paraffin sections with scale bars of 200 µm.

Tubular collagen grafts were fabricated under sterile conditions, utilizing liquid type I bovine collagen (5 mg/mL, Symatese, F). 8.5 mL of collagen solution were added to 0.8 mL of 10 × MEM and neutralized with 1 M NaOH (approx. 1.85 mL). The neutralized solution was set in a mold previously used for manufacturing tubular constructs of 3 mm inner diameter^10^. To fabricate the LD-graft, the gelated collagen was manually compressed by rolling it on filter paper, followed by an air-drying step (Fig. 1A). To fabricate the HD-graft, only air-drying was applied to achieve a desired liquid content of 1% w/w (Fig. 1A). Monitoring of the water content of collagen gels/grafts were done with a balance (Mettler Toledo, CH). The fabricated grafts were kept in PBS supplemented with 1% Penicillin/Streptomycin (Gibco, CH) and 2.5 mg/mL Fungizone (Gibco) until used.

### Scanning electron microscopy

The samples were fixed with 1% tannic acid and 1.25% glutaraldehyde, then washed with 0.1 M cacodylate, and dehydrated in increasing ethanol concentrations prior to critical point drying. Thereafter coated with gold/palladium and imaged at a voltage of 10 kV using a scanning electron microscope (SEM, XLF30, Philips).

### Mechanical evaluation

Burst pressure, ultimate tensile strength (UTS) and Young’s modulus of grafts (N = 4 in each group) were measured with an electronic manometer (Extech instruments HD750) or an Instron tensile machine (Norwood, MA, USA) as described previously^10^.

### Surgical implantation of the graft and evaluation in rabbits

Following approval by the Animal Ethics Committees of the Canton of Vaud (Authorization number: VD-2740), Switzerland and of the Faculty of Medicine of the University of Malaya (Authorization number: 2013-07-19/SUR/R/TCR), Kuala Lumpur the experiments were performed on New Zealand white male rabbits (2.5–3.5 kg; Charles River Laboratories France, and Harlan and Bred, Singapore) in Lausanne and Kuala Lumpur. All experiments were done in compliance with national directives for the care and use of laboratory animals. The surgical steps and post-operative monitoring were done as previously described^10^. Rabbits from the long-term study group were involved in the in-house breeding program of the Animal Experimental Unit of the Faculty of Medicine, University Malaya.

### Cysto-urethrography

Animals were submitted under general anesthesia for macroscopic evaluation and voiding cysto-urethrography (Visipaque 270 mg/mL). All images were collected with a Philips BV Pulsera. The diameter of the urethra was measured utilizing a scale. Knowing that the graft was sutured at 0.5 cm proximal to the base of the glans and it measured 2 cm in length, the position of the graft could be determined on the radioscopic images. It was then possible to estimate the potential presence of stenosis at the anastomotic sites. Stenosis was defined as a persistent 50% reduction of the diameter of the urethra at the same location.

### Histology and immunohistochemistry

Entire penises were harvested and fixed in 4% PFA. Specimens were embedded in paraffin, and 8 µm thick sections were prepared. Antibodies used: Mouse anti-α-smooth muscle actin (1:150, Abcam, CH), Goat anti-uroplakin-2 (1:150, Labforce, CH), Mouse anti-caldesmon (1:400, Sigma, D), Rabbit anti-CD31 (1:100, Abcam, CH), Donkey anti-mouse Alexa 647 (1:800, Abcam, CH), and Donkey anti-goat Alexa 546 (1:800, Abcam, CH). Images were taken with a Leica DM5500 microscope (Leica, D) and with a LSM 700 microscope (Zeiss, D). Alpha smooth muscle actin (α-SMA) expression was quantified with Fiji imaging program (ImageJ) as previously described^10^.

### Statistical analysis

Statistical analyses were performed using Prism v5.0a (GraphPad), using the test mentioned in the figure legends. A p-value of less than 0.05 was considered significant. All error bars in diagrams represent the standard deviation (SD).

### Data Availability

The datasets generated during and/or analyzed during the current study are available from the corresponding author on reasonable request.

## Results

### Manufacture of HD- and LD-grafts

**Figure 2:**
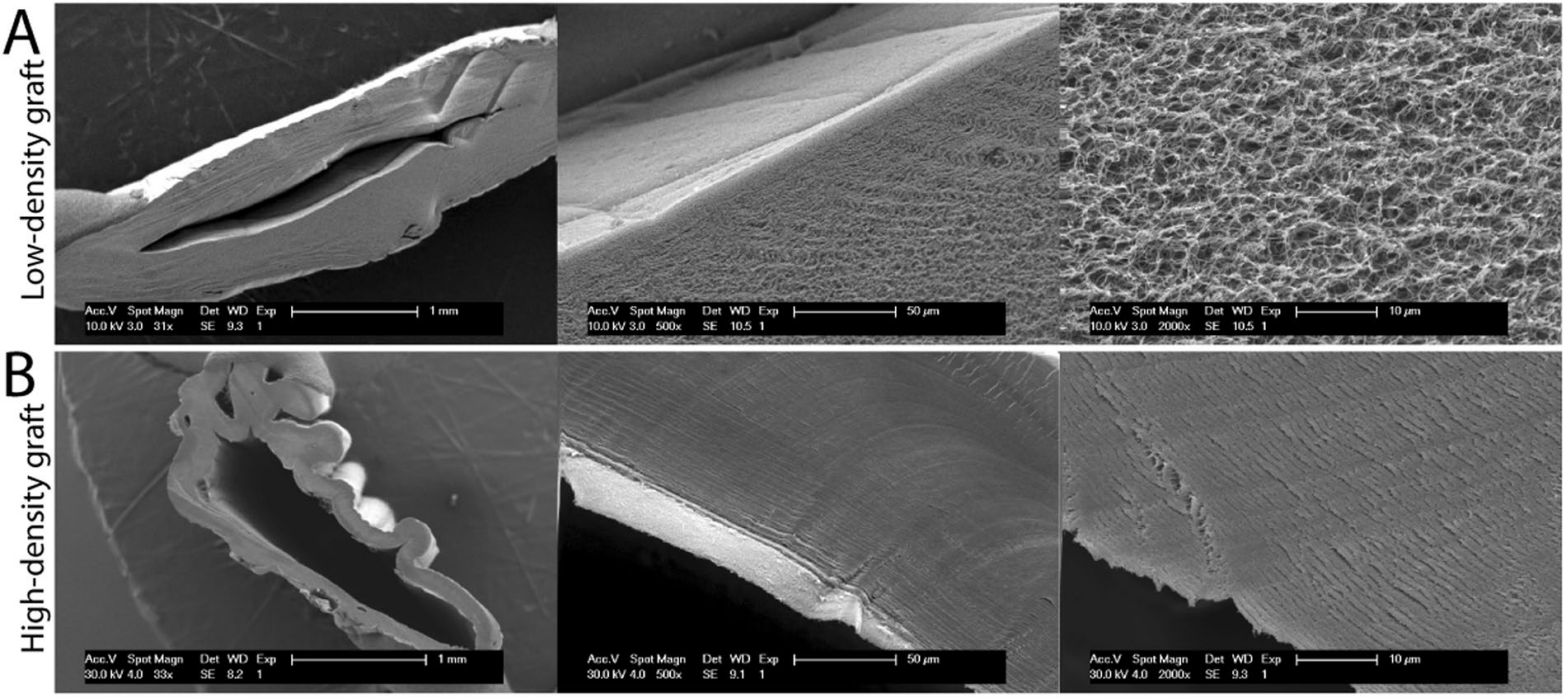
Description and characterization of manufactured collagen graft with controlled spatial orientation. (**A** and **B**) SEM images of LD- and HD-grafts with three different magnifications.

By applying a fast compression step, resulting in a less than 50% liquid loss followed by a slow air-drying step, we could control the liquid content of our produced grafts and therefore also the collagen grafts final density (Fig. 1B). This procedure yields a controlled polarized collagen fiber distribution, with a more dense structure at the compressed outer surfaces while the internal luminal part of the collagen tubular wall was left with a less dense collagen structure as shown by SEM (Fig. 2A). This graft hereafter is referred to as the LD-graft. In comparison, in the only air-dried collagen grafts, the latter become highly dense and with a uniform non-polarized collagen structure (Fig. 2B). This graft is hereafter referred to as the HD-graft. The denser HD-grafts showed significantly better mechanical properties, in terms of measured burst pressure, UTS and Young’s Modules compared to the LD-graft (Supplementary Figure 1).

### Short-term rabbit evaluation of implanted HD- and LD-grafts

**Figure 3:**
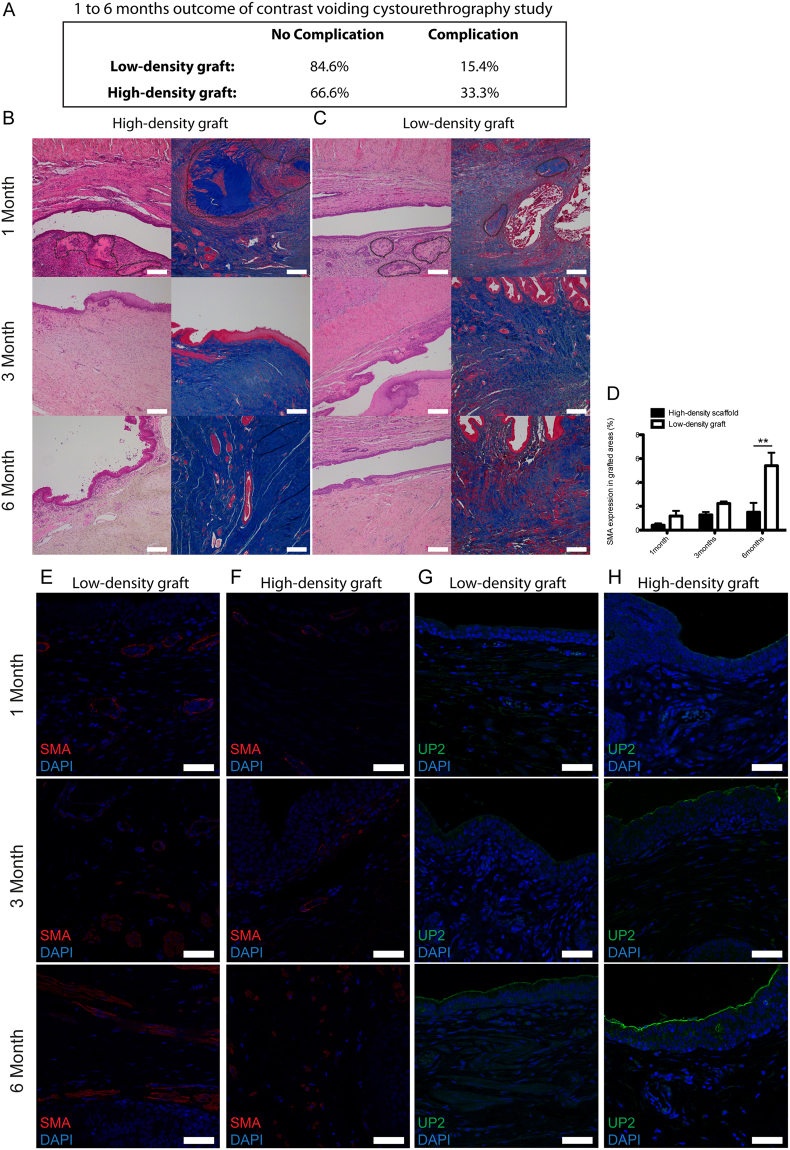
*In vivo* evaluation of high-density (HD) and low-density (LD) collagen grafts in a rabbit urethral defect model. (**A**) Functional surgical outcome analyzed by micturating cysto-urethrography of rabbit implanted with LD- and HD-grafts for 1, 3 and 6 months. (**B**) H&E and MT stained sections of the grafted area of HD-graft 1, 3 and 6 months after implantation. (**C**) H&E and MT stained sections of the grafted area of low-density graft 1, 3 and 6 months after implantation. (**D**) Quantification of SMA expression in grafted areas for rabbits implanted with LD- and HD-graft after 1, 3, and 6 months. (**E**) Immunohistochemistry for compared to the HD-grafts-SMA of LD-graft implanted for 1, 3 and 6 months. (**F**) Immunohistochemistry for SMA of HD-graft implanted for 1, 3 and 6 months. (**G**) Immunohistochemistry for Uroplakin-2 (Up2) of LD-graft implanted for 1, 3 and 6 months. (**H**) Immunohistochemistry for Up2 of HD-graft implanted for 1, 3 and 6 months. Note: areas circled in dashed black line indicating the remaining collagen pieces of the grafts after 1 month (Scale bar H&E and MT 250 µm, Scale bar SMA and Up-2 50 µm). (Error bars represent the standard deviation of four independent samples. **p < 0.01, Student t-test).

The LD-grafts were associated with a fistula formation rate of 15.4% (2 out of 13 rabbits) compared to one of 33% (3 out of 9 rabbits) in the HD-graft (Fig. 3A). No urethral stricture formation was observed. No mortality was seen as a result of graft implantation. At 1 month, it was noticed that the HD-graft still had not been completely remodelled by the surrounding tissue as shown by the persistance of implanted collagen visualized by Hemotyxlin & Eosin (H&E) and Masson’s Trichrome (MT) staining (Fig. 3B). In LD-grafts, the remaining collagen could be identified to a lesser extent in the histological samples at 1 month (Fig. 3C). The luminal side of both grafts had shown overgrowth of urothelial cells within the first month of implantation (Fig. 3B,C). Pronounced ingrowth of smooth muscle cells was still lacking at 1 month, irresepective of the graft type (Fig. 3B,C). The urothelial cells had covered the grafted region in the HD-graft at 3 months with an organized multilayered urothelial cell distribution (Fig. 3B). This was still lacking in the LD-graft at 3 months (Fig. 3C). At 6 months, the urothelium, irrespective of the graft type, showed normal stratification (Fig. 3B,C). H&E and MT stained sections showed signs of vascular structures already at 1 and 3 months in both grafts, this was confirmed by immunohistochemistry for a vascular marker, CD31 (Supplementary Figure 2). More smooth muscle cell ingrowth from the native tissue into the LD-graft was seen at 3 and 6 months when compared to the HD-graft (Fig. 3B,C). Image analyis of regenerated smooth muscle, using anti-α-smooth muscle actin (α-SMA) antibodies, confirmed the difference between the two grafts to be significant (Fig. 3D,F). A second smooth muscle cell marker, caldesmon, confirmed the α-SMA result of smooth muscle cell ingrowth (Supplementary Figure 3). Uroplakin-2, a specific antibody for terminal urothelial differentiation, also confirmed an earlier differentiation of urothelium in the HD-graft compared to the LD-graft area at 1 and 3 months (Fig. 3G,H). At 6 months, the LD-graft also showed normal Uroplakin-2 expression.

### Long-term rabbit evaluation of implanted LD-grafts

**Figure 4:**
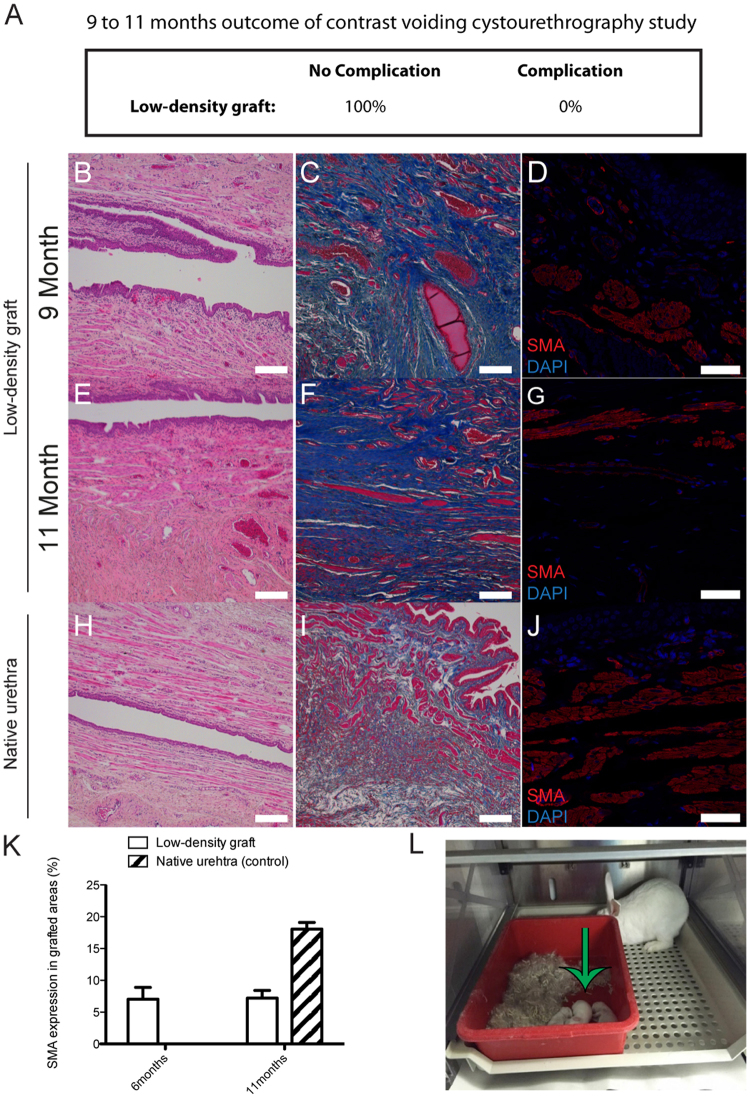
Long-term *in vivo* evaluation of low-density collagen grafts in a rabbit urethral defect model. (**A**) Functional surgical outcome analyzed by micturating cysto-urethrography of rabbits with an artificially created, circumferential urethral defect that was bridged with a LD-graft (N = 5 rabbits for 9 months, N = 2 rabbits for 11 months). (**B**,**C**,**E**,**F**, and **H**,**I**) H&E and MT stained sections of LD-graft implanted for 9 and 11 months, and a control native rabbit urethra. (**D**,**G** and **J**) Immunohistochemistry for α-SMA of LD-graft implanted for 9 and 11 months, and a control native rabbit urethra. (**K**) Quantification of SMA expression in LD-graft compared to a control native rabbit urethra. (**L**) A photo of rabbit offspring from fathers implanted with a LD-graft. Note: green arrow pointing at offspring (Scale bar H&E and MT 250 µm, Scale bar SMA 50 µm). Error bars represent the standard deviation of four independent samples.

LD-grafts were implanted in 5 rabbits for 9 months and 2 rabbits for 11 months. No complications were noted in these rabbits (Fig. 4A). At 9 and 11 months, the urothelial layer appeared comparable to the native rabbit urethra (Figs 4B,E and H) and there was pronounced ingrowth of smooth muscle cells, however, the appearance was not comparable to native rabbit urethra (Figs 4C,D,F,G,I and K). All animals in the long-term group were able to mate normally and had produced offspring (Fig. 4L).

## Discussion

In order to answer the question if collagen fiber density of a graft influences the urethral regeneration *in vivo*, we had to develop two fabrication methods to produce both a polarized low- density and a uniform high-density collagen graft. Our rabbit results show that the low-density graft had an improved smooth muscle regeneration compared to the implanted high-density graft.

Previous studies have shown that fluid content or collagen fiber density relates to the stiffness and influences material properties of the collagen gels^9,10^. The more extracted fluid from the collagen gels, the stiffer and more mechanical strong the collagen material gets. We experienced the same for our two grafts, where the surgeons reported that the HD-graft was more mechanically strong compared to the LD-graft. The HD-graft was designed to serve as a control, to represent a dense and stiff matrix (approximately 0.1 MPa), although it was not in the similar high range as the well studied SIS grafts (Small-intestine submucosa, approximately 7.2 MPa)^11^, it was still two times stiffer than the LD-graft. Importantly, the SIS is reported to have been tried with patients for urethral reconstruction but has failed and is therefor not recommended to use in urethral reconstruction^12^. According to literature the native rabbit urethra seems to have a stiffness of approximately 0.2 MPa, which is more in the range of our engineered scaffolds as compared to the SIS urethral scaffolds^13^.

We would like to challenge the current opinion in urethral reconstructive surgery that a cell-free graft is ineffective to bridge a urethral defect, and to rephrase this statement to: A cell-free graft, which is too dense or stiff, is ineffective for urethral defect grafting. Published studies have already demonstrated the failure of using stiff cell-free grafts in this clinical indication^12^. However, when these graft are seeded with cells they have shown promising result in pre-clinical models but ultimately only limited success in clinical applications^3–5,14–17^. In our graft implantation study in rabbits, the LD-graft exhibited more ingrowth of smooth muscle cells with formation of muscle bundles, due to its less dense collagen structure or, whereas the HD-graft demonstrated faster urothelial regeneration due to its denser collagen structure. This confirms similar reports where denser/stiffer grafts have been implanted in animals and showed good urothelial regeneration but has lacked an adequate smooth muscle regeneration^18^. The accelerated ingrowth of smooth muscle cells into the low-density grafts is likely due to the lower surface area of collagen that needs to be degraded and remodeled by the infiltrating cells. Examining closer the HD-graft, it showed limited smooth muscle cell ingrowth in the center of grafted areas, but a normal muscle bundle structure was observed at the interface of the graft and native tissue (Supplementary Figure 3F and G). Both implanted grafts showed no signs of urine erosion. This is most likely due to the fact that all grafts showed early urothelial regeneration protecting the underlying graft. Interestingly, the onset of fully differentiated urothelium, is delayed in the less dense grafts compared to the highly dense collagen grafts. It can be speculated that this difference in density influencing the “mechanical niche” favoring either urothelial or smooth muscle cell growth.

Improved smooth muscle regeneration is an advantage, as the primary pathology of urethral stricture is due to muscular fibrosis^19^. By 6 months, the LD-graft had significantly more expression of α-SMA compared to the HD-grafts. At 11 months, cystourethrography showed urethral patency and normal urine flow and bladder voiding. Successful mating resulting in progeny could also be seen proving clinical relevant functional regeneration of the grafted areas.

Our study has limitations. The rabbit model used had an artificially created urethral defect, with healthy urethral tissue on the edges. The graft was implanted to replace a totally excised urethral segment, what is not always mandatory as in clinical practice only partial circumferential replacement is often performed^20,21^. Although the grafts were prepared under sterile conditions, the production needs to be transferred into a GMP setting to allow translation of this technology to human application. This translation is allowed if the bioburden is lower than the sterility assurance levels set by governing authorities. To further reduce the bioburden, final sterilization can be attempted, however, it is known that sterilization can change the material properties of the grafts which might compromise the final outcome of the graft^22^.

An off-the-shelf urethral graft without the incorporation of cells and growth factors, showing adequate urothelial and smooth muscle cell ingrowth into the implanted graft, represents an important pre-clinical breakthrough. This was demonstrated by the comparable encouraging results of experimental studies conducted in two research centers in Switzerland and Malaysia opening the way for clinical translation of this cost effective, cell-free grafting for application in urethral reconstruction.

## Electronic supplementary material

Supplementary material
