# Supplementary material for: Fiber density of collagen grafts impacts rabbit urethral regeneration
Source: Sci Rep. 2018 Jul 3;8:10057. doi: 10.1038/s41598-018-27621-9 (PMC6030124; doi:10.1038/s41598-018-27621-9)
Supplement: Supplementary file 1 — Supplementary material [file 41598_2018_27621_MOESM1_ESM.pdf]

Fiber density of collagen grafts impacts rabbit urethral regeneration

HM Larsson[1,2,a], G Vythilingam[1,3,a], K Pinnagoda[1,2], E Vardar[1,2], EM Engelhardt[1], S Sothilingam[3], Rajendrarao C. Thambidorai[3], T Kamarul[4], JA Hubbell[1,5], and P Frey[1*]

[1] Institute of Bioengineering, École Polytechnique Fédérale de Lausanne, Switzerland. [2] Department of Pediatrics, Centre Hospitalier Universitaire Vaudois (CHUV), Lausanne, Switzerland. [3]Department of Surgery, University Malaya, Kuala Lumpur, Malaysia. [4] Tissue Engineering Group, Department of Orthopaedic Surgery,(NOCERAL) University Malaya Kuala Lumpur, Malaysia. [5]Institute for Molecular Engineering, University of Chicago, Chicago, IL, USA.

Supplementary information:

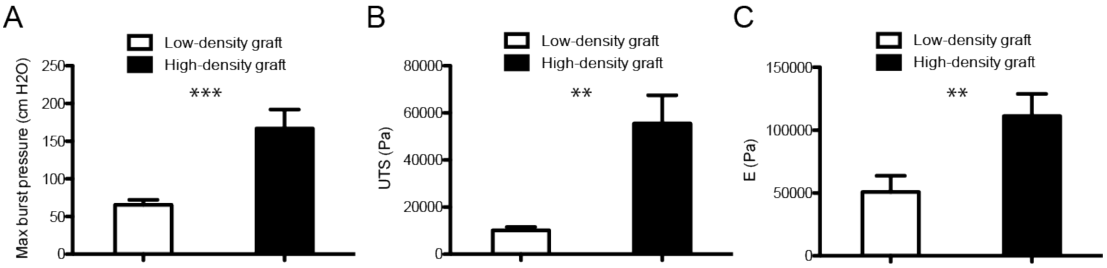

Supplementary Figure 1. Mechanical analysis of the LD and HD-grafts. (A, B and C) Burst pressures, UTS and Young's Modulus of low- and high-density grafts. (Error bars represent the standard deviation of four independent samples. **p < 0.01, ***p <0.001, Student t-test).

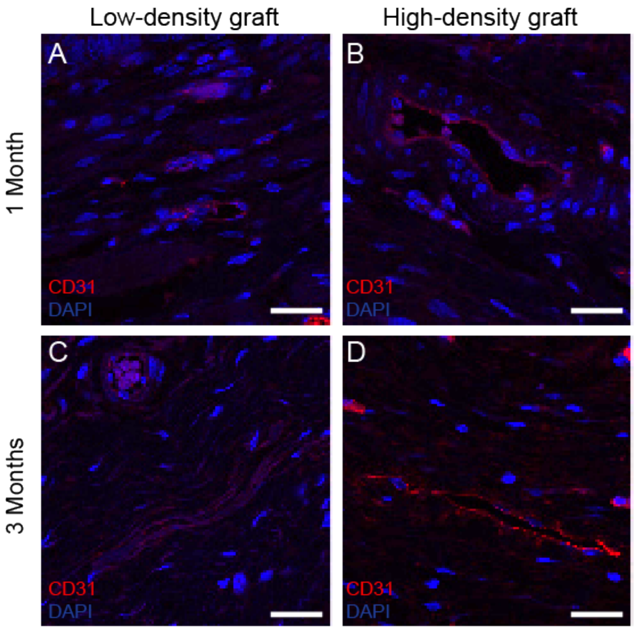

Supplementary Figure 2. Immunohistochemistry of LD- and HD-graft vascular cell marker of CD31. (A and B) Images of CD31 stained sections inside the grafted areas of rabbits implanted with LD- and HD grafts for 1 month. (C and D) Images of CD31 stained sections inside the grafted areas of rabbits implanted with LD- and HD grafts for 3 month. Scale bar 25μm

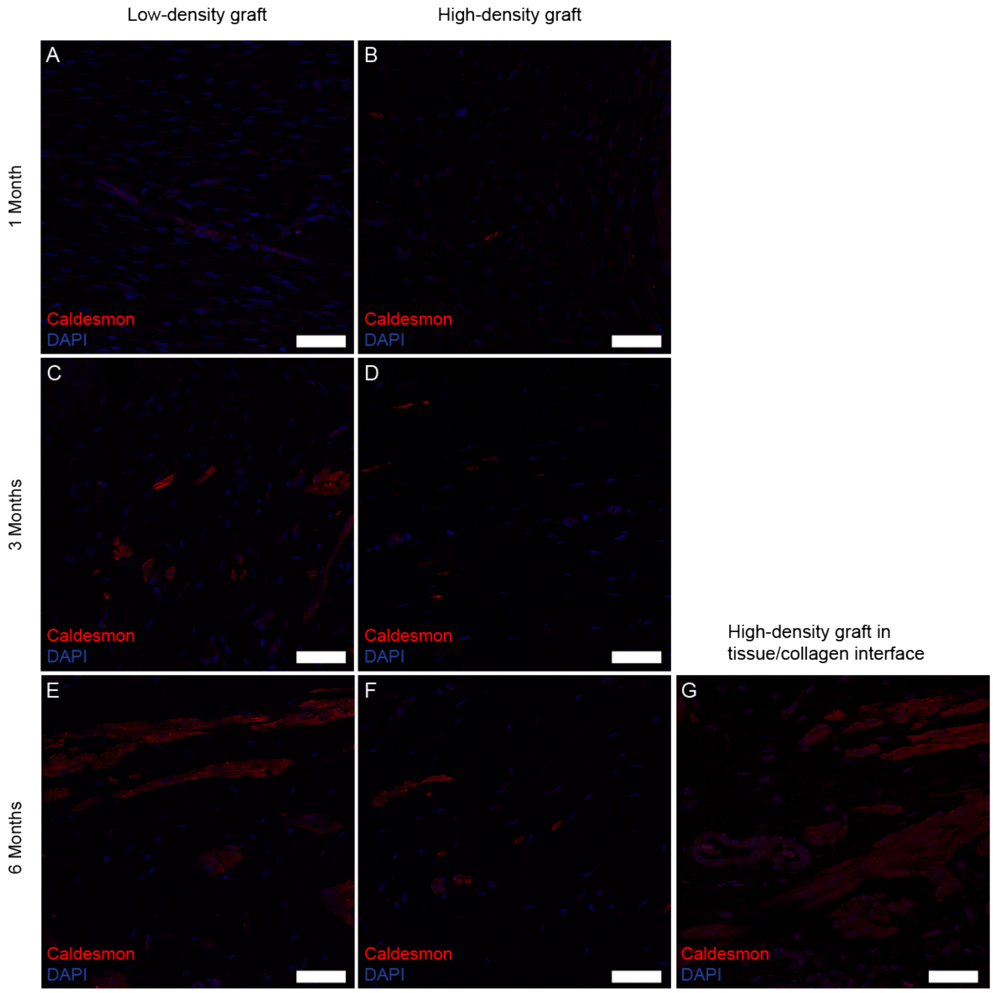

Supplementary Figure 3. Immunohistochemistry of LD- and HD-graft smooth muscle cell marker of caldesmon. (A and B) Images of caldesmon stained sections inside the grafted areas of rabbits implanted with LD- and HD grafts for 1 month. (C and D) Images of caldesmon stained sections inside the grafted areas of rabbits implanted with LD- and HD grafts for 3 month. (E and F) Images of caldesmon stained sections inside the grafted areas of rabbits implanted with LD- and HD grafts for 6 month. (G) Image of caldesmon stained section in the tissue/collagen interface of rabbits implanted with the HD grafts for 6 month. Scale bar 50µm.
